# Lieut. Harold Greene Porter

**Published:** 1918-11

**Authors:** 


**Lieut. Harold Greene Porter,** Assistant Surgeon, U. S. N. R. F., Syracuse, N. Y.; University of Syracuse, N. Y., 1901; aged 25; a Fellow of the American Medical Association; on duty at the Naval Hospital, Chelsea, Mass.; died recently from influenza.

